# Wildlife Responses to Drone Noise: A Preliminary Approach for Quantifying Disturbance During Single‐ and Dual‐Drone Flights

**DOI:** 10.1002/ece3.73643

**Published:** 2026-05-13

**Authors:** Saadia Afridi, Lucie Laporte‐Devylder, Guy Maalouf, Samuel G. Penny, Magnus Wahlberg, Blair R. Costelloe, Ulrik Pagh Schultz Lundquist

**Affiliations:** ^1^ University of Southern Denmark Odense M Denmark; ^2^ Performance Engineer ‐ Drone Noise Specialist Amsterdam the Netherlands; ^3^ Marine Biological Research Centre University of Southern Denmark Odense Denmark; ^4^ Institute of Conservation Science and Learning Bristol Zoological Society Bristol UK; ^5^ Department of Collective Behavior Max Planck Institute of Animal Behavior Konstanz Germany

**Keywords:** acoustic disturbance, conservation technology, drone wildlife monitoring, dual‐drone operations, in situ noise measurement

## Abstract

Drones are increasingly being deployed to study wildlife; however, growing evidence indicates that drone noise can alter animal behavior and confound ecological data. Few studies have quantified how acoustic exposure affects behavioral responses of wildlife. In this preliminary, descriptive study, we present a field‐based approach that integrates in situ acoustic monitoring with behavioral scoring to document group‐level disturbance patterns in free‐ranging plains zebras. In Ol Pejeta Conservancy, Kenya, we monitored how flight altitude and single vs. dual drone configurations affected plains zebras ( *Equus quagga* ) in single‐species and mixed‐species contexts. We conducted these flights between 75 and 20 m altitude with two DJI quadcopter drones (Mavic 3Pro and 3T). Drone noise was characterized using a ground‐level linear microphone array, and observers used a detailed ethogram to log vigilance and escape behaviors. Drone noise generally declined with increasing flight altitude, while dual‐drone operations introduced a disproportionate increase in acoustic exposure. This increase was non‐linear, reflecting overlapping rotor harmonics and an extended acoustic exposure window from two sequentially approaching platforms, rather than a simple doubling of noise. Bootstrapping of behavioral scores showed a directional effect, with dual‐drone flights eliciting stronger disturbance responses on average, though these estimates should be interpreted cautiously given the small sample size. Behavioral responses appeared to vary with flight altitude and group composition, with greater disturbance recorded during dual‐drone operations and in mixed‐species assemblages. These findings suggest initial, context‐specific behavioral indicators for plains zebras in open savannah conditions. Coupling in situ acoustic measurements with behavioral observations may enable a richer description of drone disturbance, though observed differences likely reflect responses to combined acoustic and visual cues rather than acoustic exposure in isolation. As a preliminary investigation, the results suggest this integrated approach can inform future field study design and evaluation of emerging configurations such as multi‐drone flights, while underscoring the need for larger‐sample validation across species and environments.

## Graphical abstract

Single drone (a) and dual‐drone (a + b) setups used to assess behavioral responses to drone noise exposure.

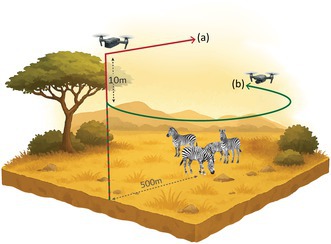

## 1 Introduction

Over the past decade, drones have become integral tools in animal research, offering a cost‐effective, low‐risk, and logistically efficient alternative to traditional monitoring methods (Elmore et al. 2023; Pedrazzi et al. 2025). Wildlife researchers commonly utilize drones to acquire data for ecological monitoring by conducting wildlife population surveys, habitat mapping, behavioral monitoring, and animal health and condition assessment, enabling observation of animals within their natural environments. However, the growing use of drones in wildlife research raises important ethical and practical concerns about potential disturbances to animals (Afridi et al. 2025; Laborie et al. 2021; Brisson‐Curadeau et al. 2024).

A growing body of empirical work shows that noise from drone rotors can elevate stress levels, provoke behavioral changes, and compromise overall animal welfare (Mesquita et al. 2022; Scobie and Hugenholtz 2016; Schwieterman 2019). Such behavioral and physiological changes can skew population counts and records of natural activity when animals alter movement patterns or display stress‐induced behaviors in response to drone overflights (Ditmer et al. 2015; Shannon et al. 2016; Scholten et al. 2020; Vas et al. 2015). While disturbance typically correlates with an observable behavioral change (Afridi et al. 2025), in some situations animals may experience a physiological stress response without externally obvious cues (Ditmer et al. 2015). However, given the difficulties in obtaining real‐time physiological data from wild or free‐roaming animals, behavioral responses are often the only indicators available for evaluating the impacts of anthropogenic stressors on animal welfare (Afridi et al. 2025). Additionally, auditory sensitivity varies widely across taxa, meaning disturbance thresholds can differ greatly between species. Earlier work has focused on methodological frameworks linking drone sound pressure level (SPL, where higher values indicate louder sound) measurements with species‐specific hearing sensitivity. Duporge et al. (2021) recorded drone SPLs under controlled conditions and applied species audiograms to evaluate perceptual masking and derive advisable flight altitudes. This perceptual weighting method provides valuable theoretical guidance, but it does not incorporate in situ behavioral responses.

Studies examining drone noise effects on wildlife can be broadly categorized by how they couple acoustic measurements with behavioral observations. Assessing these effects in situ is important precisely because sound propagation is strongly shaped by vegetation, terrain, wind, and atmospheric conditions, which can substantially alter both amplitude and spectral content (Harris 1966; ElSharkawy et al. 2024)—meaning that controlled measurements may not reflect the acoustic environment animals actually experience in the field. Yet conducting such assessments with free‐ranging wildlife is logistically challenging, as animal behavior is unpredictable and ethical constraints limit the number of controlled exposures possible. Many studies do not measure drone noise at all, attributing behavioral responses directly to drone presence without acoustic quantification (Bennitt et al. 2019; Barnas et al. 2018). Others infer noise levels from manufacturer specifications or distance attenuation models rather than direct measurement (Rümmler et al. 2016). Even studies that do directly measure drone noise often do so under conditions decoupled from the behavioral observations—different locations, times, or controlled environments—creating a mismatch between the quantified acoustic stimulus and what animals actually experienced (Mesquita et al. 2022; Duporge et al. 2021). A further limitation arises even when noise and behavior are measured at the same location and time: if the conditions are not representative of the animals' natural environment—for example, open fields rather than heterogeneous habitat with vegetation, terrain variation, wind, and atmospheric gradients—the acoustic characterization may still not reflect what animals actually hear in the field (Harris 1966; ElSharkawy et al. 2024). Taken together, these limitations point to the need for studies that measure both drone noise and behavioral responses under the same naturalistic field conditions, ensuring that the acoustic characterization directly reflects the stimulus the animals were exposed to.

While studies of animal responses to drones have focused on single drone approaches and simple overhead flights (Afridi et al. 2025; Rümmler et al. 2016), recent acoustic characterizations have shown that flight maneuvers, drone model type, and multi‐drone configurations can substantially alter both the amplitude and frequency spectrum of emitted noise (Macke et al. 2024; Zamponi et al. 2025; Schäffer et al. 2021). Multi‐UAV operations are increasingly used in wildlife surveys and conservation monitoring (Kabir and Lee 2021; Rolland et al. 2025, 2026), yet the combined acoustic footprint of multi‐drone operations has not yet been evaluated in situ in the context of wildlife behavioral research. Furthermore, several emerging drone applications—including individual animal identification, body condition assessment, and multi‐view behavioral tracking—inherently require close‐range, low‐altitude approaches that cannot be achieved with high‐altitude survey flights, even using zoom‐capable sensors (Kline et al. 2025; Jones et al. 2023). Quantifying the disturbance potential of such close‐range operations is therefore essential to ensure these workflows remain ethically viable. These findings highlight the potential for maneuver‐specific disturbance effects and the requirement for accompanying behavioral data if the implications for wildlife are to be understood.

Building on this, we paired in situ acoustic recordings with systematic behavioral observations, documenting concurrent associations between SPL, frequency distribution, and observable behavioral responses under realistic field conditions. We examined multiple flight altitudes, and both single‐ and dual‐drone operations. To our knowledge, dual drones have not previously been tested acoustically in wildlife behavioral research. Our approach integrates calibrated, in situ multi‐microphone acoustic measurements with behavioral scoring of free‐ranging wildlife. Using plains zebras ( *Equus quagga* ) in both single‐species and mixed‐species group contexts in the Kenyan savannah, we present a practical operational approach for linking sound exposure to observable behavior under real field conditions.

Given the documented relationships between acoustic exposure and behavioral disturbance, the known influence of group composition on socially mediated vigilance, and the absence of in situ multi‐drone behavioral data, we tested three hypotheses: (1) lower‐altitude flights would result in stronger vigilance and displacement responses, (2) dual‐drone operations would increase both SPLs and behavioral response intensity due to non‐linear acoustic interactions, including overlapping rotor harmonics and increased spatial complexity of the sound field, and (3) behavioral responses would be stronger in mixed‐species assemblages than in single‐species zebra groups, reflecting socially mediated vigilance in the presence of heterospecifics.

## 2 Methodology

### 2.1 Study Site

The study was conducted at Ol Pejeta Conservancy, located in Laikipia County, Kenya. The conservancy has an intact community of African wildlife species, making it particularly suitable for studying the behavioral responses of large mammals, such as plains zebras. Animals at Ol Pejeta are regularly exposed to vehicle‐based safari tourism and human presence, and the conservancy is situated in close proximity to both military and civilian airfields, resulting in relatively high levels of aviation traffic overhead. Drones are periodically deployed at the conservancy for media and communication purposes, though not in regular or intensive use. Furthermore, as the behavioral trials were conducted as part of a larger multi‐team field campaign (Lundquist et al. 2026), animals may have been exposed to drone activity by other research teams in the days prior to the trials. The extent to which any of these prior exposures influenced baseline responsiveness to drone noise is unknown and should be considered when interpreting the behavioral responses reported here.

The fieldwork at Ol Pejeta Conservancy took place during a short 10‐day campaign in which multiple research teams collected data simultaneously for different WildDrone work packages (Lundquist et al. 2026). Flight opportunities therefore had to be coordinated across teams, and the number of controlled trials that could be carried out was limited by available time, logistics, and the ethical requirement to avoid unnecessary disturbance.

Behavioral response studies were carried out on the southern plains, an open savannah region that allowed clear paths for drone flights, minimal acoustic interference, and maximum visibility for observing animals. These conditions enabled precise measurements of both drone‐generated noise and animal behavior. Focal groups were selected opportunistically during vehicle transects along established tracks within the southern plains. The first suitable group of plains zebras encountered was selected for each trial, with preference given to larger, stable groups that appeared relaxed and were predominantly grazing or stationary at the time of approach. No strict minimum group size was applied, though groups were required to contain sufficient individuals for meaningful group‐level behavioral scoring. Groups were typically located between 100 and 400 m from the nearest track, and drone flights were launched from a horizontal distance of approximately 500 m from the focal group, a distance considered sufficient to avoid pre‐flight disturbance from launch noise and activity.

### 2.2 Drone Specifications and Experimental Flights

We used two off‐the‐shelf quadcopter drones, a DJI Mavic 3Pro and a DJI Mavic 3T. DJI quadcopter platforms are among the most widely used drones in wildlife research and conservation monitoring, with DJI models having been deployed across a broad range of species and habitats (Elmore et al. 2023; Koger et al. 2023), making findings from these platforms directly relevant to a large community of practitioners. To reduce any pre‐flight disturbance (Acoustics 1993, 1996), drones were launched from a point approximately 500 m away from the focal animals. Flights were conducted by one Remote Pilot in Command and one assistant pilot, supported by a dedicated ground risk observer, an air risk observer, and a behavioral observer monitoring animal responses. All pilots held EASA A1/A2/A3 certifications. All drone operations were conducted in strict compliance with Kenyan aviation regulations, with permissions obtained from the Kenya Civil Aviation Authority (KCAA). Since Ol Pejeta Conservancy lies within military‐controlled airspace, operations were also approved by the Kenya Air Force, and real‐time coordination with military Air Traffic Control was maintained during flights. Flights were limited to 120 m AGL and operated within a 2 km radius, under Line of Sight where possible. Because drones were launched ≥ 500 m from focal animals, parts of the flight could not be kept in visual line of sight from the launch position, so BVLOS authorization was required (Maalouf et al. 2025).

**FIGURE 1:**
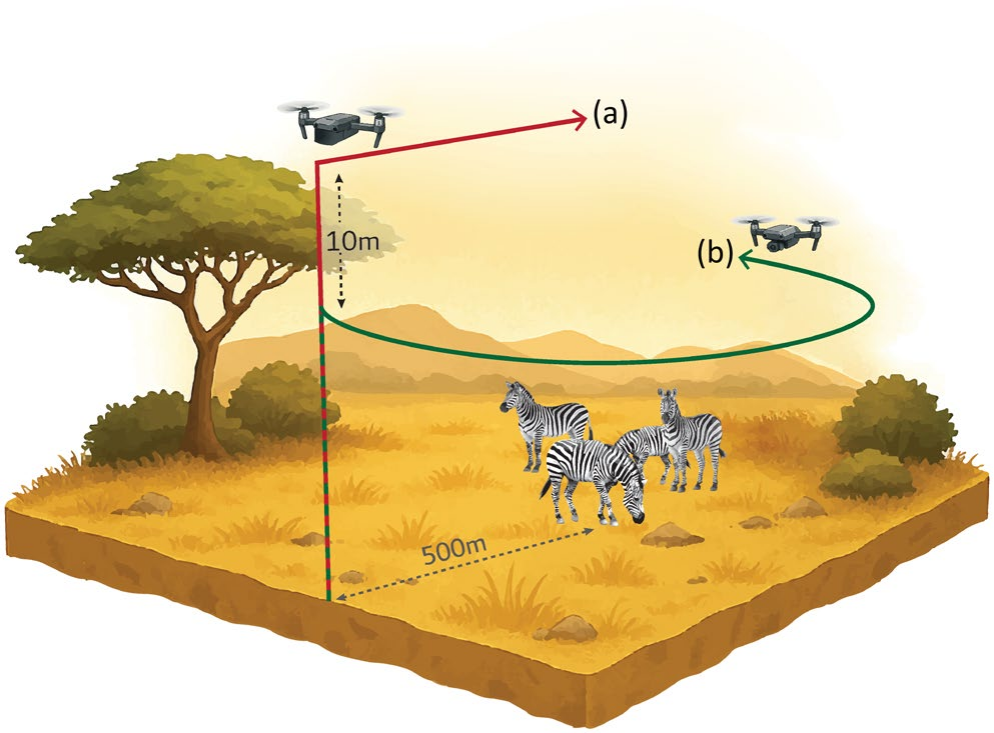
Schematic representation of the single‐drone (a) and dual‐drone (a + b) flight setups used to assess behavioral responses to drone noise exposure. In the single‐drone setup (a), one drone approached the focal animal group directly overhead from a launch point approximately 500 m away, climbing to the required altitude before proceeding horizontally at 3–5 m/s. In the dual‐drone setup (a + b), a second drone (b) followed a lateral arc trajectory above the animal group simultaneously, maintaining a fixed vertical separation of 10 m from the first drone. Both setups included a hover phase of up to 60 s directly above the focal group before returning to the launch point. The schematic illustrates the relative positions of the two drones, the 10 m vertical separation, and the 500 m horizontal distance from the launch point to the focal animals.

For each flight, one drone climbed vertically to the required altitude, then proceeded horizontally toward the animals at approximately 3–5 m/s. Upon reaching a position directly above the group, the drone hovered in place up to 60 s and flew back along the original trajectory to the launch site (Figure 1). For dual‐drone flights, a second drone was launched simultaneously and approached the group along a lateral arc path, maintaining a fixed vertical separation of 10 m from the first drone. After each altitude trial, the drone landed and a brief pause of approximately 90–120 s occurred before ascending to the next height. This pause was primarily for checking GPS stability and battery levels, rather than for resetting animal behavior, meaning that some carryover of alertness or habituation between sequential flights cannot be fully ruled out. This process was repeated at different altitudes depending on the experimental configuration (Table 1). The two drones followed different approach paths (direct overhead vs. lateral arc) to reflect realistic operational scenarios; as a result, platform differences and trajectory effects are partially confounded and are interpreted here as integrated exposure conditions rather than isolated model‐specific effects.

**TABLE 1:** Flight configurations, drone models, and species observed per flight. Species composition reflects animals encountered in the field during each trial and is included here for contextual reference; group size and species identity varied across flights due to the ad‐hoc focal‐animal sampling approach.

Flight no.	Experimental configuration	Drone model	Species observed
1	30 m direct approach; hovering up to 60 s	Mavic 3T	Zebras (9) & Giraffes (2)
2	45 m direct approach; hovering up to 60 s	Mavic 3T	Zebras (7) & Giraffes (2)
3	60 m direct approach; hovering up to 60 s	Mavic 3T	Zebras (8)
4	75 m direct approach; hovering up to 60 s	Mavic 3T	Zebras (10 adults, 1 calf) & Thomson's gazelles (12)
5	30 m direct approach; hovering up to 60 s	Mavic 3Pro	Zebras (21)
6	45 m direct approach; hovering up to 60 s	Mavic 3Pro	Zebras (10)
7	60 m direct approach; hovering up to 60 s	Mavic 3Pro	Zebras (18)
8	75 m direct approach; hovering up to 60 s	Mavic 3Pro	Zebras (14)
9	Dual drones: 3Pro at 30 m direct, 3 T at 20 m arc approach; hovering up to 60 s	Mavic 3T, Mavic 3Pro	Zebras (9)
10	Dual drones: 3Pro at 40 m direct, 3T at 30 m arc approach; hovering up to 60 s	Mavic 3 T, Mavic 3Pro	Zebras (16)
11	Dual drones: 3Pro at 50 m direct, 3T at 40 m arc approach; hovering up to 60 s	Mavic 3 T, Mavic 3Pro	Zebras (11) & Thomson's gazelles (28)
12	Dual drones: 3Pro at 60 m direct, 3T at 50 m arc approach; hovering up to 60 s	Mavic 3T, Mavic 3Pro	Zebras (32)
13	Dual drones: 3Pro at 70 m direct, 3T at 60 m arc approach; hovering up to 60 s	Mavic 3T, Mavic 3Pro	Zebras (17) & Giraffes (7)

We employed two experimental setups using an ad‐hoc focal‐animal sampling approach. In the single‐drone setup, for each drone platform, we identified one focal group and conducted four sequential flights at 75, 60, 45, and 30 m AGL (configuration 1–8 in Table 1). At each altitude, the drone approached the group, hovered up to 60 s, then landed before ascending to the next height. This design enabled controlled comparison of behavioral responses and acoustic exposure across vertical distances. In total, we performed four flights per platform, with a mean flight duration of 3 min (take‐off to landing).

In the dual‐drone setup, two drones were flown simultaneously over a single focal group. In total, two focal groups were used across the dual‐drone trials: for the first group, we executed three sequential flights corresponding to configurations 9–11 (Table 1); for the second group, we executed two sequential flights corresponding to configurations 12–13 (Table 1). The Mavic 3Pro followed the same direct overhead trajectory as during single‐drone flights, whereas the Mavic 3T followed a lateral arc above the animal group. Five altitude combinations were tested: (20 m, 30 m), (30 m, 40 m), (40 m, 50 m), (50 m, 60 m), and (60 m, 70 m). The two drones had a fixed vertical separation of 10 m in all combinations to examine the combined and stacked effect of noise exposure. The altitude increments used in dual‐drone flights differ from those in single‐drone flights because the dual‐drone design prioritized examining the interaction between simultaneously operating platforms at closely spaced altitudes rather than direct comparability with single‐drone conditions. This represents a design limitation and direct altitude‐matched comparisons between single‐ and dual‐drone conditions should therefore not be made. At each altitude pair, both drones approached their prescribed altitudes, hovered up to 60 s, then landed. In total, we performed five paired flights, with a mean flight duration of 6 min (take‐off to landing). Dual‐drone flights were longer because synchronizing two platforms required additional time for climb, altitude stabilization, and coordinated landing procedures. However, the hover period experienced by the animals remained 60 s in both setups, meaning that most behavioral exposure occurred during comparable time windows. Nevertheless, animals were exposed to drone noise and visual stimuli during the approach and departure phases as well, and the longer total flight duration of dual‐drone operations means this cumulative exposure cannot be fully separated from the effect of operating two drones simultaneously. As a result, direct comparisons of behavioral responses between single‐ and dual‐drone treatments should be interpreted with caution, as observed differences may partly reflect greater cumulative exposure duration in dual‐drone trials rather than the effect of simultaneous dual‐drone operation alone.

### 2.3 Behavioral Observation and Analysis

In mixed‐species savannah systems, behavioral responses to anthropogenic stimuli are often socially mediated. Herbivores frequently monitor both conspecifics and heterospecifics, and group‐level vigilance can propagate through social information transfer rather than direct perception of the stimulus alone (Palmer and Gross 2018; Meise et al. 2018). Such socially amplified responses are well documented in ungulates and may shape how disturbance manifests at the group level (Kitchen et al. 2010; Beauchamp et al. 2021). Given this, and the practical constraints of fieldwork with free‐ranging wildlife, the present study adopts a conservative group‐level, ordinal behavioral scoring approach. Widely used in disturbance ecology (Beale 2007), this draws on established behavioral response categories—spanning undisturbed behavior, vigilance, displacement, and escape—documented across aircraft and drone‐wildlife disturbance research (Mesquita et al. 2022; Calef et al. 1976; Goldstein et al. 2005; van Vuuren et al. 2023). The resulting six‐level ordinal scale (0–5), described in Table 2, operationalizes these categories into a numbered framework for systematic scoring across flight trials. As a group‐level measure, however, this approach does not capture within‐group variation in individual responses, individual‐level behavioral differences, or temporal dynamics within a flight phase—a limitation that directly affects the resolution of the analysis.

**TABLE 2:** Ethogram defining behavioral responses and associated disturbance levels (0: No/minimal disturbance to 5: Severe disturbance) observed in zebras and giraffes during drone exposure.

Behavior	Definition	Score
Undisturbed	Calm standing or lying; minor within‐group movement; normal grazing without visible alertness or interruption of activity	0
Observant	Brief head‐lifts to scan surroundings, then resumes prior activity	1
Vigilance	Sustained alertness with head orientation toward drone or raised head with eyes above shoulder height	2
Low‐intensity displacement	Slow walking or gradual repositioning away from the drone without coordinated retreat	3
High‐intensity displacement	Coordinated group retreat, trotting, or rapid relocation indicating elevated avoidance behavior	4
Flushing	Sudden, panic‐driven flight from area, signaling strong disturbance	5

Behavioral observations were carried out during wildlife flight trials from a research vehicle positioned at distances (≥ 500 m) sufficient to minimize observer disturbance (Acoustics 1993, 1996), focusing on plains zebra groups in both single‐species and mixed‐species assemblages. Animal behaviors were continuously logged using an ethogram (see Table 2) during every flight. The ethogram defined six ordered disturbance levels (0–5), ranging from no observable disturbance (0) to severe disturbance (5). Score 0 corresponded to undisturbed behavior, including calm standing or lying, minor within‐group movement, or normal grazing without visible alertness. Score 1 indicated brief observant behavior, such as short head‐lifts to scan the surroundings followed by a return to prior activity. Score 2 represented sustained vigilance, defined as prolonged head orientation toward the drone, tense posture, or persistent scanning behavior without relocation. Displacement behaviors were separated into two distinct categories to differentiate intensity. Score 3 corresponded to low‐intensity displacement, defined as slow walking or gradual repositioning away from the drone without coordinated retreat. Score 4 corresponded to high‐intensity displacement, defined as coordinated group retreat, trotting, or rapid relocation indicating elevated avoidance behavior. Score 5 represented flushing behavior characterized by sudden, panic‐driven flight from the area. For each flight phase, behaviors were classified using a predefined decision rule to ensure consistency and objectivity. The assigned behavioral disturbance score reflected the highest sustained behavioral intensity observed in the majority (> 50%) of individuals within the focal group during that phase, rather than averaging responses across individuals. Brief or isolated reactions exhibited by only a small number of individuals were not used to determine the final group‐level score. If behavioral intensity escalated during a single flight phase (e.g., sustained vigilance followed by displacement), the higher sustained intensity level was recorded. In cases where two distinct behavioral intensities were sequentially sustained for comparable durations within the same flight phase, a range (e.g., 2–3) was reported to reflect this progression. Ranges therefore indicate temporal escalation of group‐level responses rather than variation among individuals.

Behaviors were logged in real time using field datasheets and cross‐checked against simultaneous video clip recordings to confirm the accuracy of observations. Video recordings were reviewed manually by the same observer rather than processed through dedicated behavioral annotation software. This approach was considered appropriate given the group‐level, ordinal nature of the scoring framework, which did not require frame‐by‐frame temporal precision. All behavioral scoring was conducted by a single trained observer. If animal behavior escalated and showed evasive behavior or signs of distress, drone operations were immediately stopped to ensure the welfare of animals in each experiment. The low sample size (*n* = 13 flight trials) reflects the difficulty of finding and exposing suitable groups of free‐ranging animals to experimental stimuli, coupled with the need to prevent undue stress. The analyses presented here are exploratory and descriptive in nature and do not aim to provide inferential statistical testing. Given the small number of flight trials and the multiple sources of variation across flights (drone model, altitude, flight path, species composition, and group size), formal statistical modeling was not attempted. With only four independent focal groups and substantial variation in confounding factors across trials, any model would be severely underpowered and its outputs unreliable. Instead, we complemented descriptive behavioral scoring with quantitative summaries, effect sizes, and nonparametric bootstrap resampling to evaluate the robustness and directionality of observed patterns. We acknowledge that this precludes formal inference and that future studies with larger, more controlled datasets should explore ordinal mixed‐effects or permutation‐based modeling frameworks to quantify the relative contributions of altitude, drone configuration, and species composition to disturbance responses.

### 2.4 Acoustic Measurement Setup and Procedures

**FIGURE 2:**
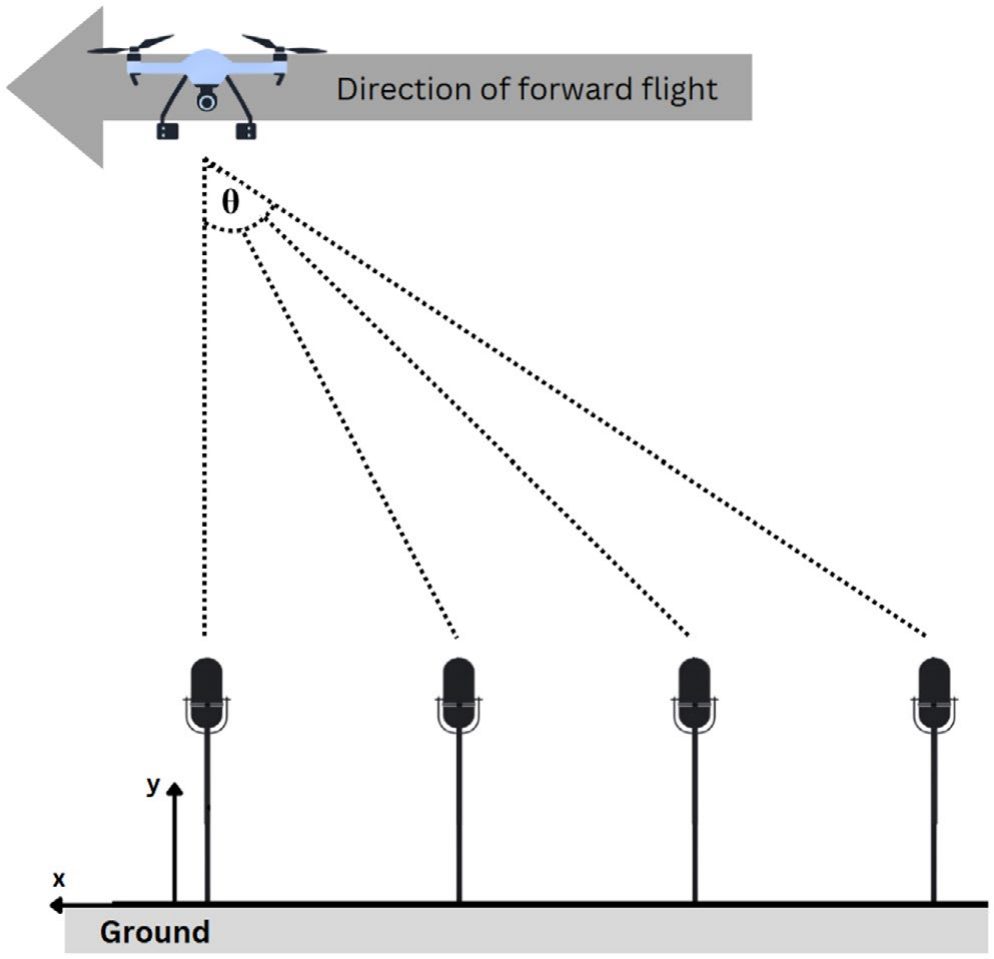
Schematic of the drone noise measurement setup during forward flight, showing the drone's path over ground microphones. The angle θ indicates the line‐of‐sight to each microphone for analyzing noise propagation.

A linear microphone array was positioned on the ground, parallel to the drone flight path to measure drone‐generated noise. The drone‐generated noise was recorded using two Bruel & Kjaer 4966 condenser microphones and two AKG C214 microphones. All microphones were pre‐calibrated using a 1 kHz, 94 dB test tone generator, thus guaranteeing the accuracy of the signal and inter‐channel consistency throughout the recordings. Because the Brüel & Kjær 4966 microphones provide measurement‐grade frequency responses suitable for quantitative acoustic analysis, all SPL and spectral analyses were conducted exclusively using the B&K microphones. The AKG C214 microphones were retained as supplementary audio reference channels but were not used for quantitative calculations. The signals were recorded using the Zoom H6 at 48 kHz sampling frequency and 32‐bit resolution, suitable for capturing broadband acoustic signals relevant to drone noise characterization. SPL values were derived retrospectively from the calibrated audio recordings using MATLAB, rather than measured directly in the field with a sound level meter. It should be noted that retrospective SPL reconstruction introduces uncertainty relative to in situ calibrated measurements taken simultaneously during behavioral trials, as environmental conditions such as wind speed, ambient noise, and microphone positioning may have varied between sessions. Z‐weighting (unweighted) was applied to preserve the full frequency spectrum of the recorded signal, rather than A‐weighting which emphasizes frequencies most relevant to human hearing. This approach was chosen to enable detailed post hoc spectral analysis and to allow direct comparison of acoustic signatures across flight configurations. The resulting SPL values serve as the acoustic stimulus descriptor characterizing the noise produced during flight configurations comparable to those used in the wildlife trials. The array was positioned near the observation vehicle, approximately 500 m from the focal animal groups and in the vicinity of the drone launch point. Each microphone was spaced at 5 m intervals along the axis parallel to the drone route (see Figure 2). Recording procedures were guided by the standards outlined in ISO 3746:2010 (International Organization for Standardization 2010). Microphones were treated as independent measurement points with fixed spacings, orientation, and gain levels maintained between sessions. Windshields were employed on all microphones to reduce wind‐induced noise contamination. Recordings were made under stable weather conditions with wind speeds below 5 m/s. Ambient sounds were recorded during each session to ensure that background noise levels were low enough to permit precise measurement of drone noise. Because only the B&K microphones were used for quantitative analyses, the influence of cross‐microphone variability was minimized; however, the presence of different microphone models in the array is acknowledged as a minor source of potential variability.

To avoid any influence of the microphones on wildlife behavior, the acoustic measurements were collected only after the completion of the wildlife flights. Following the behavioral trials, the microphone array was set up in the same open‐field environment. The drone was then flown directly over the array to record acoustic signatures under comparable environmental conditions without influencing animal behavior. This setup allowed the acoustic measurements to represent the same field context and flight types used during the wildlife trials while ensuring that microphone placement and the field crew did not affect animal behavior.

## 3 Results

### 3.1 Acoustic Emission of Drone Flights

**FIGURE 3:**
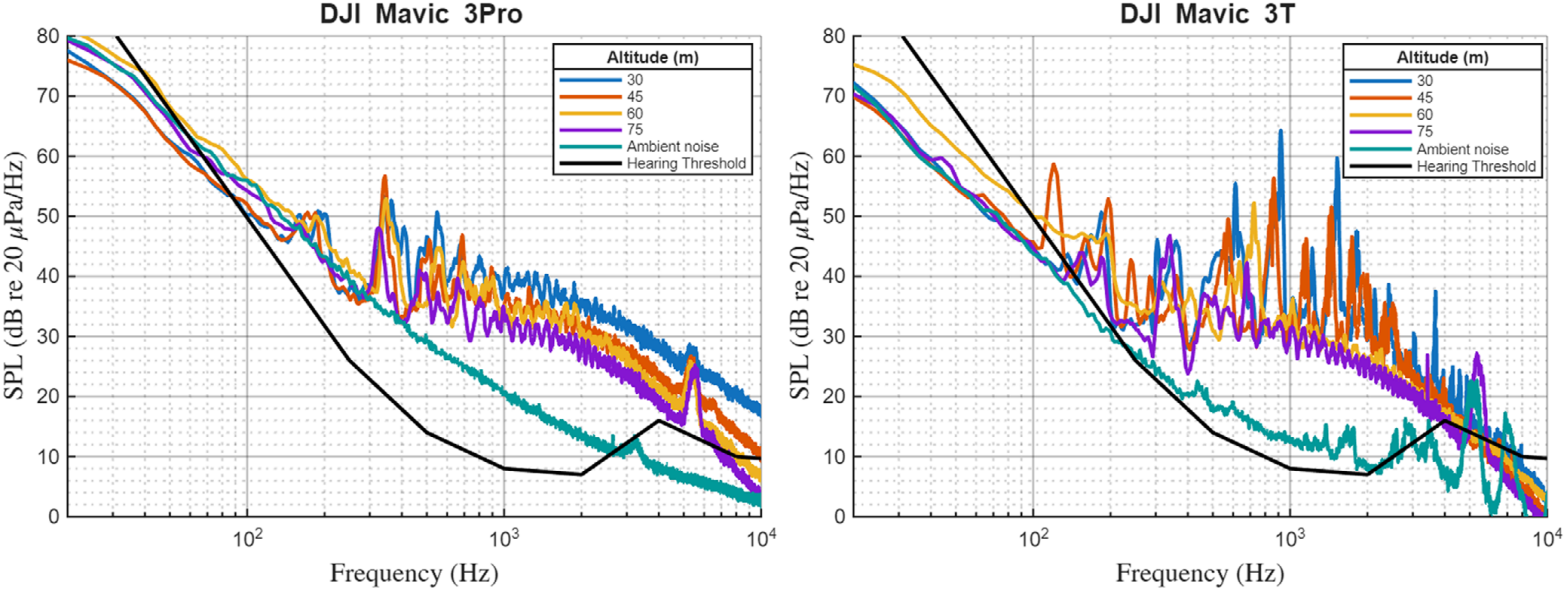
Frequency‐amplitude profiles recorded by a low‐sensitivity microphone at varying drone flight altitudes. The graph illustrates the variation in noise amplitude across frequencies as a function of drone altitude, with lower altitudes generally exhibiting higher amplitude values.

Noise recordings during flight trials showed a consistent trend of increasing SPL with decreasing altitude. During hovering at low altitude, drone‐generated noise consistently attained greater SPL than at higher altitude. The highest SPL during a single phase of drone operation reached approximately 75 dB re 20 μPa at 20 m range. These trends were seen repeatedly for both drones, demonstrating a clear relationship between flight altitude (i.e., distance from the receiver) and measured SPL. Changes in SPL related to altitude were also evident in the horizontal flight trials regardless of speed (3–5 m/s). The lower the altitude of drone flights (below 30 m), the higher the recorded noise levels. Spectral analysis (Figure 3) indicated altitude‐dependent variations in acoustic signatures depending on specific drone maneuvers. Hover phases comprised strong low‐frequency tones (<400 Hz) of the acoustic spectrum. These tonal features relate to some fundamental blade‐passing frequencies and low‐order harmonics and are generated when the rotors create noise at a higher thrust level. In the case of forward‐flight operation, however, the spectrum is more distributed toward the medium‐frequency band (400–800 Hz) due to a fairly uniform rotor‐blade loading under continuous forward thrust. The two platforms differed in configuration, Mavic 3 Pro flew in stock configuration while Mavic 3 T flew with a small auxiliary hand‐hold bracket to aid safe hand launching and catching in the field. This additional fitting changes the local fuselage geometry and thus perhaps changes rotor inflow or scatters body‐borne noise; coupled with differences in camera/gimbal mass and center of gravity, this may well affect rotor RPM requirements and raise the broadband energy in low‐mid bands. Hence, platform differences should remain qualitative in their interpretation.

**FIGURE 4:**
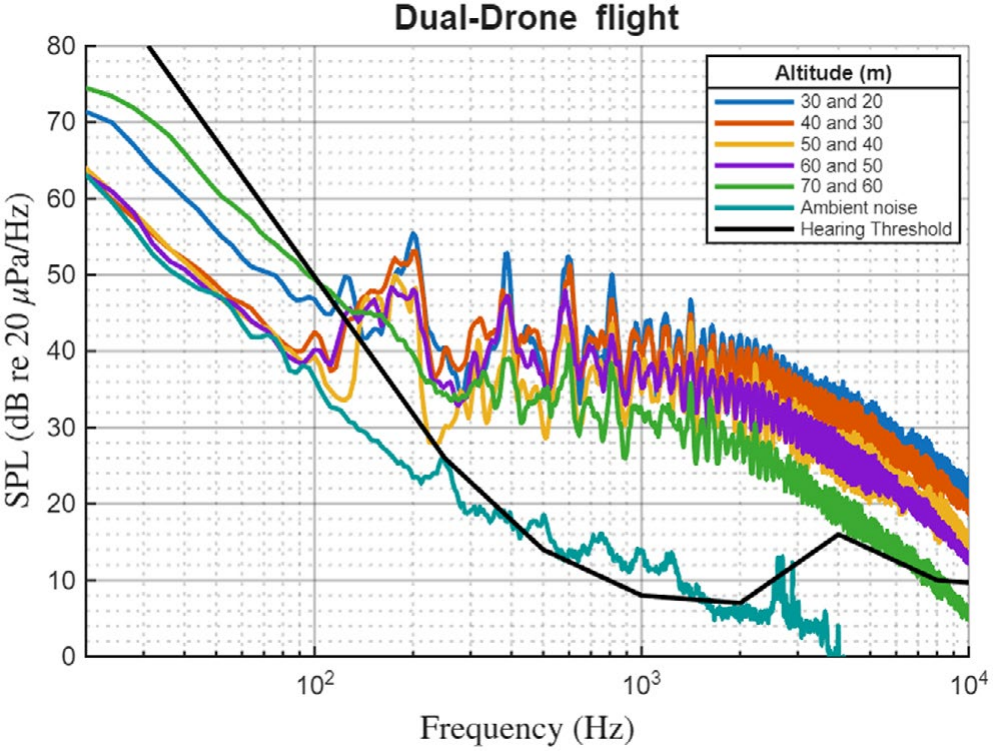
Frequency‐amplitude profiles at varying dual‐drone flight altitudes. The hearing threshold curve (black dashed line) represents horse ( *Equus caballus* ) audiometric data from Fay (Fay 1988), used as a phylogenetic proxy for zebra auditory sensitivity.

Operating two drones resulted in distinct acoustic signal patterns and an added complexity (i.e., acoustic superposition). In the dual‐drone portion of the study, measured SPL during two‐drone flight conditions was higher (5–8 dB) than single‐drone flight, and the duration of exposure above a given SPL threshold was longer on average, reflecting the sequential approach of two platforms rather than a simple doubling of noise (Figure 4). In addition, the tonal features of the two‐drone operating modes had audible overlaps accounting for the effective extent of drone noise and overall sound pitch. Beyond amplitude enhancement, the two drones sequentially approach each other, lengthening the temporal duration of acoustic exposure. The overlap in rotor harmonics from both drones resulted in the emergence of a broader frequency band, mostly from 200 Hz to 800 Hz. This frequency range falls within the sensitive auditory range of large terrestrial mammals (Stansbury et al. 2025), suggesting potential perceptibility across this band.

**FIGURE 5:**
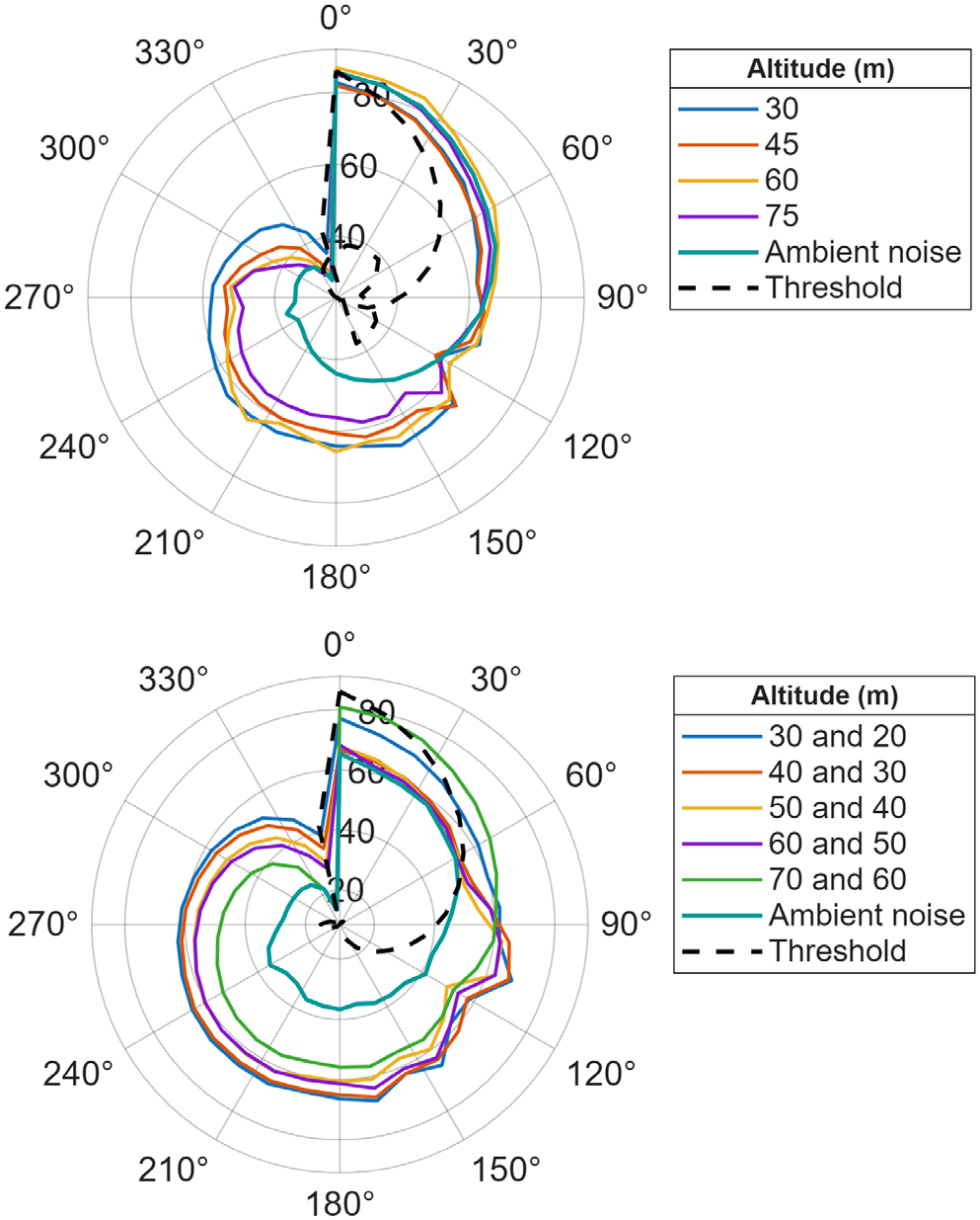
Polar representation of 1/3‐octave‐band sound pressure levels measured for single‐ (top) and dual‐drone (bottom) flights, overlaid with ambient noise and animal hearing threshold. The hearing threshold curve (black dashed line) represents horse ( *Equus caballus* ) audiometric data from Fay (Fay 1988), used as a phylogenetic proxy for zebra auditory sensitivity given the absence of a published zebra audiogram.

The spatial distribution of drone noise plays a fundamental role in disturbance ecology beyond basic sound amplitude and spectral characteristics. Polar plots identify how frequency specific noise levels (at 3‐octave‐band SPL) vary with flight altitude (Figure 5). Each colored trace corresponds to a measurement at a particular altitude; the radial axis indicates SPL, and the angular axis orders the center frequencies clockwise from low to high. At low altitudes, the lower‐frequency arc of the plot, corresponding to bands around 200–1000 Hz, exhibits a broad plateau of elevated levels at low altitudes, with maxima around 75 dB re 20 μPa. With increasing altitude, the SPLs across the same angular sector decrease by 10–15 dB, producing a more compact profile, while higher‐frequency bands show smaller relative changes. An overlaid auditory threshold curve provides context for perceptibility across angles, highlighting that the greatest overlap with mammalian hearing occurs in the mid‐to‐low frequency arc. These angular–spectral patterns show how altitude shifts the distribution of acoustic energy across frequency bands. The hearing threshold curve overlaid on Figures 3, 4, 5 is based on horse ( *Equus caballus* ) audiometric data from Fay (Fay 1988), used as a phylogenetic proxy for zebra auditory sensitivity given the absence of a published zebra audiogram. Because each altitude condition was recorded in a single pass, within‐condition variability measures could not be derived and are therefore not included in the polar plots.

### 3.2 Behavioral Responses of Plains Zebras

**FIGURE 6:**
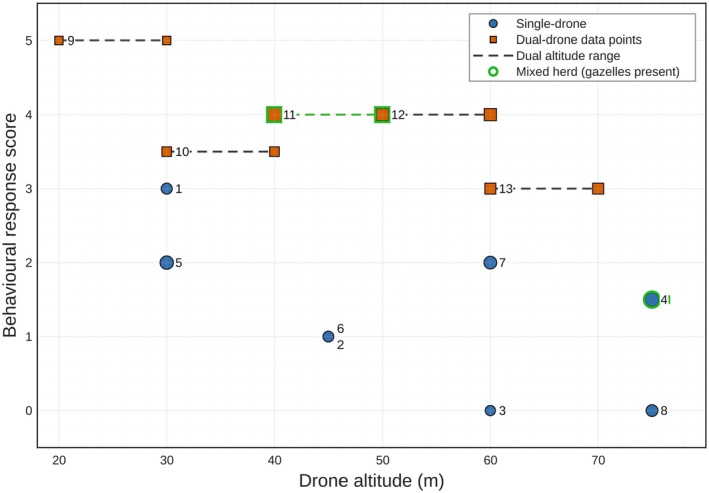
Behavioral responses to drone altitude in single‐ and dual‐drone trials. Single‐drone trials cluster at low disturbance scores and show a clear decline in response with increasing altitude. Dual‐drone trials form a separate band of consistently high scores across the entire altitude range.

**FIGURE 7:**
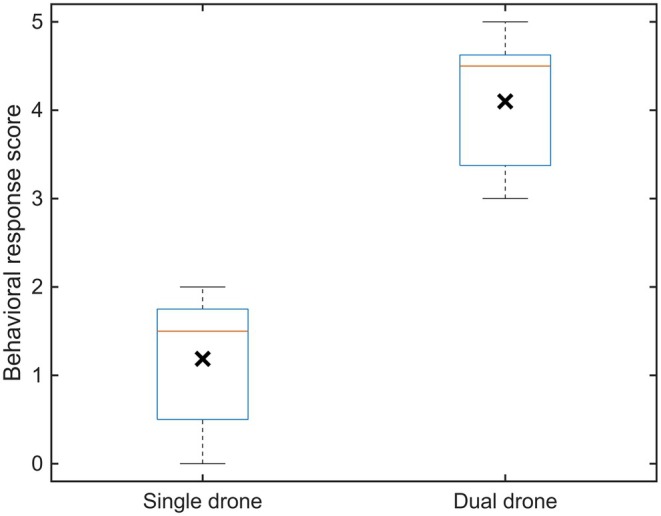
Distribution of behavioral disturbance scores for single‐ and dual‐drone flights. Boxplots show medians (red horizontal lines) and interquartile ranges; whiskers indicate the data range. Black X markers indicate mean scores, consistent with the bootstrap analysis based on mean differences.

Behavioral disturbance scores varied with flight altitude and drone configuration across 13 flight trials (Table 3, Figure 6). Behavioral disturbance scores (Table 4) showed consistently low responses during single‐drone flights (mean ± SD of 1.19 ± 0.73; *n* = 8) and substantially elevated responses during dual‐drone operations (mean ± SD of 4.10 ± 0.86; *n* = 5). Mean disturbance scores differed 2.91 points between single‐ and dual‐drone conditions, corresponding to a large, standardized effect size (Cohen's *d* = 3.7) and the non‐overlapping distributions indicate consistent group‐level responses (Figure 7), though the small sample size warrants caution. Bootstrap resampling (20,000 iterations) yielded a 95% confidence interval of 2.09–3.74 for the mean difference, and the difference was positive in 100% of bootstrap replicates. Given the modest sample size, these bootstrap estimates should be interpreted cautiously and taken as indicators of the direction and consistency of the effect rather than precise estimates of its magnitude. While Figure 6 illustrates altitude‐dependent patterns across individual flight trials, Figure 7 summarizes the overall distribution of disturbance scores between single‐ and dual‐drone conditions, providing context for the bootstrap analysis.

**TABLE 3:** Behavioral responses of zebras in both single‐species and mixed‐species groups to drone approaches in the Kenyan savannah. The varied species composition highlights ecological complexity and interspecific influence during drone‐induced disturbances.

Flight condition	Response before drone encounter	Response during drone encounter	Behavioral response scoring
1	Zebras grazing in small subgroups (2–3 individuals); giraffes slowly walking	Zebras showed sustained directional movement and relocated at a walking pace; increased spacing between individuals	3
2	Zebras mixed activity (grazing and standing calmly); one giraffe feeding, another moving toward vegetation	Zebras exhibited brief head lifts and mild alertness, then resumed grazing; giraffes unaffected	1
3	Zebras resting or standing relaxed in small groups	Minimal response; continued resting and standing	0
4	Both species actively grazing	Gazelles displayed immediate vigilance causing mild alertness among zebras; mother zebra moved calf away	1
5	Mixed behaviors: grazing (11), slowly walking away (8), lying down (1), alert posture (2)	Zebras monitored drone with head‐turns, resumed activity after hover	2
6	Combination of grazing and relaxed standing	Observant; occasional head‐lifting, no prolonged disturbance.	1
7	Engaged in routine grazing behavior	Zebras exhibited sustained vigilance with intermittent head‐raising and minor repositioning, without coordinated retreat; giraffes unaffected	2
8	Diverse behaviors: grazing, standing, lying down	Minimal response; normal behavior continued	0
9	Zebras slowly moving together pre‐exposure	Panic in one triggered herd‐wide running; significant disturbance	5
10	Grazing and slowly walking in two loose groups	Defensive clustering, some rapid dispersal, frequent head‐turning; maternal vigilance noted	3–4
11	Actively grazing and slowly moving	Gazelles fled, causing zebra panic; brief relocation then resumed grazing	4
12	Primarily grazing; few interacting or standing calmly	One zebra panicked, triggered herd response and dispersal; others formed tight groups or relocated	4
13	Two giraffes initially cautious, intermittently walking and pausing while observing drone; zebras loosely grouped	Giraffes moved toward zebras; zebras clustered defensively, then moved away calmly	3

**TABLE 4:** Summary statistics for behavioral disturbance scores during single‐ and dual‐drone flights.

Condition	*n* (no. of flights)	Mean	SD	Median	Range
Single drone	8	1.31	1.03	1.25	0–3
Dual drone	5	3.90	0.74	4.00	3–5

As predicted, disturbance for single‐ and dual‐drone flights also varied with altitude (Table 5, Figure 7). Single‐drone flights showed an increase in disturbance as altitude decreased. Higher altitudes corresponded to only brief vigilance by multiple individuals (60 m: mean 0.75, range 0–1.5), at intermediate altitudes (40–59 m) disturbance increased modestly (mean 1.25), while at the lowest altitudes (below 40 m), sustained vigilance and short‐distance relocation were more common (mean 2.00). Because altitude‐band subgroup sizes were very small, these values should be interpreted as descriptive patterns rather than formal statistical estimates. Even so, these patterns indicate a monotonic, altitude‐dependent response during single‐drone operations, though it should be noted that the fixed ascending altitude order means that habituation effects between sequential flights cannot be ruled out as a contributing factor. In contrast, dual‐drone flights showed consistently high disturbance across all altitudes, including at 60 m (score 3). At 40–59 m and 39 m, disturbance remained elevated (means 4.5 and 4.25, respectively), indicating that different operating procedures may be needed for multi‐drone operations. Again, given the limited number of dual‐drone passes per altitude, these values are presented as exploratory indicators of possible trends rather than precise altitude effects.

**TABLE 5:** Behavioral disturbance scores across altitude bands for (a) single‐drone and (b) dual‐drone flights. Subgroups with very small sample sizes (*n* < 3) are reported descriptively, and SD values are omitted to avoid overstating precision.

Altitude band	*n* (flights)	Mean	SD	Median	Range
(a)					
≥60 m	4	0.88	1.03	0.75	0–2
40–59 m	2	1.00	—	1.00	1–1
≤39 m	2	2.50	—	2.50	2–3

(b)					
≥60 m	1	3.00	—	3.00	3–3
40–59 m	2	4.00	—	4.00	4–4
≤39 m	2	4.25	—	4.25	3.5–5

Group composition appeared to influence behavioral responses. Plains zebras in mixed‐species assemblages containing Thomson's gazelles ( *Eudorcas thomsonii* ) showed stronger disturbance responses (scores 3–5) compared to zebras in single‐species groups (scores 0–2; Figure 6), a pattern consistent with, though not directly testing, socially mediated vigilance in heterospecific groups, and this observation should not be interpreted as a statistically supported finding. During single‐drone flights, zebras in single‐species groups displayed generally mild behavioral responses including raised heads, brief gaze direction changes, and occasional short‐distance relocation, consistent with the low disturbance scores recorded at higher altitudes. During dual‐drone operations, responses were more pronounced, with zebras displaying clustering behavior, fast pacing, trotting, and evasive movements consistent with the elevated disturbance scores recorded across all altitude bands. In mixed‐species assemblages, Thomson's gazelles were typically the first to show behavioral responses to drone noise, followed by responses among the zebras in the same group. By contrast, the presence of giraffes was not associated with similarly elevated zebra disturbance scores, with mixed‐species groups containing giraffes showing variable disturbance scores across flights.

## 4 Discussion

We present a preliminary framework that integrates calibrated in situ acoustic measurements with group‐level behavioral scoring to document initial disturbance indicators for plains zebras during drone operations in an open savannah environment. The patterns observed here are exploratory and should be interpreted as context‐specific behavioral indicators rather than established species‐wide disturbance thresholds. By pairing in situ measurements of sound with behavioral trials conducted under the same field conditions, we address a gap in the literature where acoustic and behavioral data are typically collected separately. Our approach enables direct mapping of sound pressure levels, frequency distributions, and tonal characteristics to specific flight parameters, revealing how changes in acoustic stimuli—such as low‐frequency rotor tones during hover or broader spectral signatures during forward flight—relate to behavioral responses in the study population. These findings are preliminary and context‐specific, and should be interpreted as initial behavioral indicators for plains zebras in open savannah conditions rather than established species‐wide disturbance thresholds.

These observed relationships provide preliminary indications of how acoustic characteristics may relate to behavioral thresholds in the species studied. First, they provide an empirical basis for the design of low‐noise propeller systems, particularly targeting reductions in the 200–800 Hz frequency band that overlaps with the sensitive auditory range of large mammals. Second, they inform flight path and trajectory optimization, identifying operational profiles (e.g., higher minimum altitudes, flight paths that avoid exposing animals to rear‐hemisphere noise maxima) that minimize disturbance. Third, they inform the development of evidence‐based guidelines that empower wildlife researchers to responsibly balance data collection needs with animal welfare considerations. In practice, this means that behavioral and population‐monitoring studies should consider conducting flights at altitudes above 50–60 m AGL, where disturbance responses are reduced—consistent with recommendations from previous studies of African savannah wildlife (Afridi et al. 2025; Bennitt et al. 2019). This recommended altitude range should be viewed as context‐specific to open savannah systems and the species studied here, and it should be noted that the fixed ascending altitude order used in this study means altitude‐dependent effects and habituation effects cannot be fully disentangled. Advances in camera and sensor technology enables high‐resolution data capture even from these distances (Scobie and Hugenholtz 2016; Hodgson et al. 2016; Seymour et al. 2017).

It is important to note that the behavioral responses observed in this study reflect animals' integrated perception of the drone as a multimodal stimulus, combining both acoustic and visual cues. Single‐ and dual‐drone flights differed not only in acoustic exposure but also in visual complexity, the number of moving aerial objects, and the spatial dynamics of the flight paths. The present experimental design does not allow acoustic and visual components to be disentangled, and the observed behavioral differences between single‐ and dual‐drone configurations should therefore not be attributed exclusively to acoustic properties. Future studies employing acoustic playback experiments without visual co‐factors would help isolate the specific contribution of drone noise to disturbance responses.

Plains zebras showed altitude‐dependent behavioral responses consistent with their status as a vigilant prey species in open savannah systems. During single‐drone flights over single‐species groups, responses were generally mild, comprising brief head‐lifts and occasional short‐distance relocation at lower altitudes. During dual‐drone operations, responses were more pronounced, with zebras displaying clustering behavior, fast pacing, trotting, and evasive movements, particularly at lower altitude combinations. Responses were stronger in mixed‐species assemblages, where Thomson's gazelles ( *Eudorcas thomsonii* ) were observed to respond before zebras, a pattern consistent with heterospecific vigilance cascades documented in African ungulate communities (Palmer and Gross 2018; Meise et al. 2018), though the present data do not allow this mechanism to be formally tested. By contrast, the presence of giraffes did not appear to have a similar amplifying effect on zebra responses, with mixed‐species groups containing giraffes showing variable disturbance scores across flights. The contrasting patterns observed in gazelle‐ versus giraffe‐mixed assemblages suggest that species identity may influence group‐level disturbance responses, though the mechanisms underlying these differences could not be determined from the present dataset.

The influence of mixed‐species group composition on behavioral responses warrants further consideration. Mixed‐species associations are known to facilitate heterospecific information transfer, whereby individuals monitor and respond to the vigilance and alarm signals of other species in addition to conspecifics (Palmer and Gross 2018). In the present study, gazelles initiated alarm responses before zebras, suggesting they acted as early‐warning indicators within mixed groups, consistent with the broader literature on heterospecific vigilance cascades in African ungulate communities (Palmer and Gross 2018). Group size may also have independently influenced behavioral responses. In larger groups, there is a greater likelihood of including highly sensitive individuals whose strong reactions may trigger contagion effects, potentially amplifying the overall group‐level disturbance response (Palmer and Gross 2018). The trade‐off between vigilance and foraging in group‐living organisms (Olson et al. 2015) further suggests that drone‐induced vigilance may carry indirect costs beyond immediate disturbance, including reduced foraging time. These dynamics highlight the importance of considering group composition and interspecific interactions when designing and interpreting drone‐based wildlife surveys in mixed‐species assemblages.

Beyond these qualitative patterns, the quantitative differences between single‐ and dual‐drone flights were substantial. Dual‐drone operations elicited disturbance scores that were, on average, 2.91 points higher than single‐drone flights, representing an extremely large standardized effect (Cohen's d=3.7). The 95% bootstrap confidence interval (2.09–3.74) confirmed that this difference was consistently present across resampling iterations, and the directional effect was maintained in 100% of bootstrap replicates. These results indicate that the observed patterns reflect strong and reliable group‐level responses rather than isolated events, and they underline that the number of drones in operation is a key determinant of disturbance intensity, with altitude acting as a mitigating factor only under single‐drone conditions.

The present findings are broadly consistent with existing in situ and ex‐situ work on drone disturbance in large mammals. Mesquita et al. (2022) documented megafauna responses to drone noise under controlled ex‐situ conditions, reporting species‐specific variation in disturbance intensity that aligns with the altitude‐dependent and configuration‐dependent patterns observed in the present study. van Vuuren et al. (2023) examined free‐ranging ungulate responses to UAV overflights in an African savannah reserve, finding that flight altitude and drone model were key determinants of disturbance, and that response probability decreased by approximately 20% with each subsequent pass, suggesting rapid habituation potential that could not be assessed in the present study given the limited number of trials per focal group. It is also worth noting that the fixed ascending altitude order used in this study means that the observed decrease in disturbance at higher altitudes may partially reflect habituation across sequential flights rather than altitude effects alone. Bennitt et al. (2019) similarly documented that altitude and horizontal distance were primary drivers of disturbance responses across seven African herbivore species, a pattern consistent with the altitude‐dependent responses observed here. Taylor et al. (2025) provided complementary evidence using acoustic playback experiments across three reserves in South Africa, demonstrating that free‐roaming giraffes responded more strongly to drone noise than to a natural control stimulus, and that response magnitude was modulated by prior exposure history. At a broader level, the patterns observed here are consistent with meta‐analytic evidence showing that anthropogenic noise reliably elicits behavioral responses across a wide range of taxa (Kunc and Schmidt 2019), and that chronic noise exposure can have cascading effects on wildlife communities beyond immediate disturbance (Kok et al. 2023). While the present study focuses on plains zebras in an open savannah context, comparable disturbance dynamics have been reported in other taxa including pinnipeds (Stepien et al. 2024), where flight altitude, drone model, and approach type all influenced disturbance levels, highlighting that flight configuration is a relevant determinant of disturbance across species groups.

Environmental setting is known to play an important role in shaping wildlife responses to drone disturbance. Open savannah habitats such as Ol Pejeta provide conditions that favor efficient sound transmission, with minimal vegetation‐induced scattering or absorption of acoustic energy (Marten and Marler 1977; Marten et al. 1977). This means that drone noise propagates with relatively little attenuation across the landscape, potentially increasing the detection range and perceived intensity of the acoustic stimulus. Combined with the unobstructed sightlines characteristic of open savannah, animals in this habitat may detect approaching drones earlier and at greater distances than in more structurally complex environments (Testroote 2018; Stankowich 2008). In contrast, vegetated or topographically heterogeneous areas may dampen both visual and auditory signals through scattering, absorption, and masking effects, potentially shifting disturbance thresholds upward (Marten and Marler 1977; Marten et al. 1977; Morton 1975; Chen et al. 2021). Consequently, mitigation strategies should be habitat‐specific, with open habitats requiring more conservative flight profiles, including higher minimum altitudes and restrictions on simultaneous drone operations.

Our results also emphasize the increased risks of disturbance associated with dual‐drone flights. Apart from increasing SPL, simultaneous flights created overlapping tonal components that may have been more difficult for animals to spatially localize than a single noise source. It is plausible that this more complex acoustic environment contributed to the elevated disturbance responses observed, though this interpretation is speculative given that stress levels were not directly measured and acoustic features beyond SPL and broad frequency content were not formally analyzed. To be clear, what is directly supported by the results is the observed increase in SPL and broader frequency content during dual‐drone operations; the suggestion that animals may have experienced difficulty spatially localizing overlapping noise sources remains speculative and was not formally tested. With drone advancements and increased frequency of multi‐drone operations in research and commercial arenas, these risks are expected to increase. Dual‐drone results should be interpreted cautiously because platform and trajectory differences could not be fully separated. Operational guidelines are necessary to specifically address the increased noise potential stemming from multi‐drone deployment and to offer clear criteria on when and how such flights should be performed to avoid as much disturbance as possible. Where feasible, restriction to single drones at higher altitudes would appear to minimize disturbance to mild vigilance without considerable displacement. The consistently elevated disturbance scores observed across all dual‐drone altitude combinations raise a broader question about when multi‐drone operations are justified. Researchers should weigh the scientific value of multi‐drone data collection against the potential welfare impacts and conduct preliminary single‐drone trials to establish species‐specific baseline responses before deploying multi‐drone configurations. Where multi‐drone operations are deemed necessary, they should be restricted to the highest feasible altitudes and limited in duration.

The small sample size and absence of statistical modeling represent important limitations of this study. Furthermore, the 13 flight trials were conducted across only four independent focal groups, which limits the independence of observations and should be considered when interpreting the reported patterns. Additionally, flights were conducted in a fixed ascending altitude order, meaning that altitude‐dependent effects and potential habituation effects between sequential flights cannot be fully disentangled; randomizing the altitude sequence in alternating trials is recommended in future work. Furthermore, because all behavioral scoring was conducted by a single observer, inter‐observer reliability could not be assessed. These constraints reflect the challenges of field‐based wildlife disturbance research, where ethical, logistical, and regulatory requirements limit the number of controlled exposures. While these restrictions prevented formal inference, the consistent patterns observed across independently encountered groups provide meaningful baseline evidence of how large mammals respond to single and dual drone flights. Furthermore, the use of a coarse group‐level behavioral metric means that fine‐scale behavioral differences, temporal response dynamics, and individual‐level variation cannot be resolved with the current dataset, representing an important constraint on the depth of inference possible from these observations. Our findings should therefore be interpreted as preliminary behavioral indicators rather than definitive disturbance thresholds, offering a foundation for more comprehensive future studies.

Overall, this study demonstrates the value of integrating acoustic and behavioral measurements under natural field conditions. Although focused on plains zebras within one open savannah ecosystem, the approach establishes a practical workflow that can be adapted to other species, habitats, and operational scenarios. As drone use continues to expand in conservation and ecological research, such combined methods will be essential for developing guidelines that minimize disturbance while maintaining data quality.

## 5 Future Research Work

Future work should build on the combined in situ acoustic and behavioral monitoring approach implemented here. We recommend prioritizing this framework during preliminary flight trials, particularly when testing new platforms, flight configurations, or operating in novel habitats, to establish context‐specific disturbance indicators before moving toward targeted mitigation strategies or routine survey operations.

### 5.1 Field Trials With Integrated Acoustic and Behavioral Monitoring

As this experiment focused on plains zebras in single‐species and mixed‐species group contexts in an open savannah setting—though mixed‐species herds including Thomson's gazelles were present during some trials—future experiments should attempt to assess reactions across a wider range of focal species and habitats such as forests, wetlands, and human‐modified landscapes. These field trials should incorporate the deployment of calibrated microphone arrays alongside systematic behavioral observations, enabling direct linkage between acoustic signatures (SPL, frequency distribution, directionality) and observed disturbance behaviors. Applying this framework more broadly during baseline assessments will enhance the generalizability of acoustic disturbance thresholds and provide stronger evidence for ethical drone‐use guidelines.

### 5.2 Path Planning to Minimize Acoustics

Trajectory optimization algorithms should be investigated to minimize acoustic exposure through intelligent path generation (Sarhan et al. 2025). The possibilities of changing flight altitude, angles, and speeds in real time, based on environmental and species‐specific factors, could allow researchers to reduce disturbance zones and avoid maneuvers that produce high SPL at ground level. Modeled and field‐validated adaptive flight strategies should follow to confirm their effectiveness in dynamic environments.

### 5.3 Propellers Designed to Reduce Noise

Following the acoustic profiles provided in this study, the next step involves developing low‐noise propeller designs with the goal of reducing noise emissions predominantly in the low‐frequency bands (200–800 Hz) of trajectory flight, which are most relevant to large terrestrial mammals. This should involve biomimetic design principles, aerodynamic modeling, and replicable testing using standard microphone arrays within semi‐field (controlled outdoor environments) and controlled laboratory settings, ultimately leading to quieter drone systems suitable for ecological monitoring (Wei et al. 2025; Wang and Liu 2022; Li et al. 2019).

Beyond propeller geometry, drone arm length and overall platform architecture also influence noise output. Increasing the distance between rotors by extending arm length reduces inter‐blade acoustic interactions and can lower overall noise levels. Additionally, electronic speed controller (ESC) frequencies contribute to the acoustic signature of drone platforms and can be modified through tuning, offering a further avenue for noise reduction. These hardware‐level modifications typically require custom drone builds or collaboration with manufacturers, but represent promising directions for developing purpose‐built, low‐disturbance platforms for wildlife research.

Together, these three research directions offer a coherent strategy for minimizing drone disturbance to wildlife. Applying this combined approach across species and habitats will build a more complete understanding of disturbance thresholds, while also providing the practical evidence needed to develop species‐ and context‐specific operational guidelines. By linking technological improvements with on‐the‐ground behavioral evidence, this framework ensures that the growing use of drones in ecological research can advance in a way that is both effective and ethically responsible.

## 6 Conclusion

This study developed and demonstrated a replicable integrated approach for assessing how drone noise affects animal behavior, combining calibrated in situ acoustic measurements with structured group‐level behavioral scoring. Applied to plains zebras in single‐species and mixed‐species group contexts in an open Kenyan savannah, the approach revealed altitude‐dependent and configuration‐dependent behavioral responses, with dual‐drone operations eliciting substantially stronger disturbance than single‐drone flights, mixed‐species assemblages showing stronger responses in the presence of Thomson's gazelles, a pattern consistent with, though not directly testing, heterospecific vigilance processes. By integrating these data streams under the same naturalistic field conditions, we documented concurrent associations between sound pressure levels, frequency spectra, and flight variables and observed disturbance behaviors. These associations should be interpreted as descriptive relationships rather than isolated causal links, given that acoustic and visual cues were not experimentally disentangled. This integrated approach offers a practical framework for the design of ethical, low‐disturbance drone flights and should be prioritized in future work when defining disturbance indicators for additional species, environments, or operational conditions, particularly during preliminary and exploratory flight trials. As a case study in applied interdisciplinary research, this project is intended to inform, rather than resolve, the challenge of minimizing disturbance, supporting the continued use of drones in ecological research in a way that aligns with conservation priorities and animal welfare.

## Author Contributions

**Saadia Afridi:** conceptualization (lead), data curation (lead), formal analysis (lead), methodology (lead), writing – original draft (lead), writing – review and editing (lead). **Lucie Laporte‐Devylder:** investigation (equal), writing – original draft (equal), writing – review and editing (equal). **Guy Maalouf:** writing – original draft (equal), writing – review and editing (equal). **Samuel G. Penny:** supervision (equal), validation (equal), writing – original draft (equal), writing – review and editing (equal). **Magnus Wahlberg:** supervision (equal), validation (equal), writing – review and editing (equal). **Blair R. Costelloe:** conceptualization (supporting), writing – review and editing (equal). **Ulrik Pagh Schultz Lundquist:** project administration (equal), resources (equal), supervision (equal), writing – review and editing (equal).

## Funding

This work is supported by the WildDrone MSCA Doctoral Network funded by EU Horizon Europe under grant agreement no. 101071224.

## Conflicts of Interest

Author Saadia Afridi was employed by the company Avy B.V. The remaining authors declare that the research was conducted in the absence of any commercial or financial relationships that could be construed as a potential conflicts of interest.

## Supporting information


**Data S1:** ece373643‐sup‐0001‐DataS1.

## Data Availability

All the required data are uploaded as Supporting Information.
