# Correction to “Wildlife Responses to Drone Noise: A Preliminary Approach for Quantifying Disturbance During Single‐ and Dual‐Drone Flights”

**DOI:** 10.1002/ece3.73818

**Published:** 2026-06-05

Afridi, S., L. Laporte‐Devylder, G. Maalouf, et al. 2026. “Wildlife Responses to Drone Noise: A Preliminary Approach for Quantifying Disturbance During Single‐ and Dual‐Drone Flights” *Ecology and Evolution* 16, no. 5: e73643. https://doi.org/10.1002/ece3.73643

The article was originally published incorrectly as a Nature Notes article type. The correct article type is Research Article, and the article has been updated to reflect this.

We apologize for this error.

